# Exploration of NMDA and GABA receptor-mediated plasticity induced by 10-Hz repetitive transcranial magnetic stimulation

**DOI:** 10.1038/s41398-026-04159-3

**Published:** 2026-06-17

**Authors:** Jamie Kweon, Hakjoo Kim, Prem Ganesh, Megan M. Vigne, Andrew M. Fukuda, Boyu Ren, Linda L. Carpenter, Joshua C. Brown

**Affiliations:** 1 Brain Stimulation Mechanisms Laboratory, Division of Depression and Anxiety Disorders, McLean Hospital, Belmont, MA 02478 USA; 2 Department of Psychiatry, Harvard Medical School, Boston, MA 02115 USA; 3 Department of Psychiatry and Human Behavior, Alpert Medical School of Brown University, Butler Hospital, Providence, RI 02903 USA; 4 Department of Clinical Science, Kaiser Permanente Bernard J. Tyson School of Medicine, Pasadena, US

**Keywords:** Learning and memory, Neuroscience

## Abstract

Although 10-Hz repetitive transcranial magnetic stimulation (rTMS) is an FDA-approved treatment for depression, we do not understand the mechanisms through which rTMS induces therapeutic change. Two competing theories suggest that 10-Hz rTMS induces N-methyl-D-aspartate receptor (NMDAR)-dependent long-term potentiation (LTP), and/or removal of inhibitory gamma-aminobutyric acid receptors (GABARs). We examined these two proposed mechanisms of action in the human motor cortex in a double-blind, randomized, four-arm crossover study in six healthy subjects. We tested plasticity with motor-evoked potentials (MEPs) and learning with serial reaction time task (SRTT) before and after 10-Hz rTMS in the presence of four drugs separated by 1 week each: placebo, d-cycloserine (DCS 100 mg), DCS 100 mg + NMDAR partial antagonist dextromethorphan (DXM 150 mg), and lorazepam (LZP 2.5 mg). NMDAR agonism by DCS enhanced rTMS-induced cortical excitability relative to placebo. This facilitation was specifically blocked by combining DCS with DXM. LZP administration resulted in a significant reduction of MEP amplitudes after rTMS. Results suggest that 10-Hz rTMS-induced plasticity works primarily through NMDAR dependent modulation with LTP-like mechanisms as opposed to GABAergic reduction. Our pilot study is the first to compare NMDAR v. GABAR in rTMS-induced plasticity and found the safety and feasibility of a 4-arm crossover protocol with each drug combined with the full 3000 pulse 10-Hz rTMS protocol. Our results may guide future clinical translation leveraging mechanistic understanding to improve therapeutic efficacy.

## Introduction

Repetitive transcranial magnetic stimulation (rTMS) has demonstrated remarkable therapeutic potential, but real-world effectiveness of this tool has remained unchanged, perhaps in part because clinical application has outpaced our understanding of rTMS mechanisms. Knowledge of neuronal and synaptic level mechanisms of rTMS may be necessary to optimize its clinical outcomes in brain medicine.

High-frequency rTMS (≥ 5 Hz) has been theorized to work through a form of synaptic plasticity called long-term potentiation (LTP), which is mediated by N-methyl-D-aspartate receptors (NMDARs) [1]. 10-Hz magnetic stimulation of mouse hippocampal slices showed increases in hallmark features of LTP such as dendritic spine size, α-amino-3-hydroxy-5-methyl-4-isoxazolepropionic acid receptor (AMPAR) subunit GluA1 expression, and NMDAR-dependent post-synaptic currents [2]. Curiously, the same 10-Hz stimulation protocol also decreased gamma-aminobutyric acid receptor (GABAR)-mediated inhibitory currents, GABAR synaptic expression, and associated scaffolding proteins [3], mitigating normal inhibition.

In healthy humans, NMDAR partial agonist d-cycloserine (DCS) was sufficient to enhance 10-Hz rTMS-mediated facilitation of corticomotor plasticity measured by motor-evoked potentials (MEPs) and recruitment curves [4, 5]. LTP-like effects were also observed with 10-Hz rTMS + DCS with apparent occlusion of intracortical facilitation (ICF) and potential homeostatic depression initiation captured during short-interval intracortical inhibition (SICI) [6]. These findings were supported by increased Hebbian-like plasticity in those with more motor learning [7], and by diminished plasticity in caffeine users presumably exposed to homeostatic plasticity through chronic overstimulation [8]. The role of GABARs in facilitatory rTMS protocols has been explored to a lesser extent. Though GABA_A_ agonist diazepam and GABA_B_ agonist baclofen both effectively blocked LTP-like effects of paired-associative stimulation (PAS) [9, 10], the studies did not use stimulation protocols with clinical applications and therefore do not provide insight into therapeutic rTMS mechanisms of action. GABAR antagonists have not been tested in pharmacologic modulation of rTMS protocols, possibly for safety reasons like seizure risk. A systematic review on this topic is reported elsewhere [11].

rTMS protocols are commonly referred to as “excitatory” and “inhibitory” for respective high- and low-frequency protocols; however, these short-hand rules are often inaccurate, in part due to interindividual variability, but more fundamentally due to other differing parameters within a given frequency. For example, train duration and pulse number produce opposing effects on cortical excitability within a 10-Hz and iTBS protocol, respectively [12–14]. To date, all 10-Hz corticomotor physiology protocols have differed substantially from the FDA-cleared 3000 pulse, 4 s train duration, 26 s intertrain interval protocol for depression [15, 16]. For the first time, we characterized the neurophysiologic effect of this clinically oriented 10-Hz rTMS protocol and the role of NMDARs and GABARs in those effects.

We conducted a four-arm crossover study with pharmacologic augmentation of NMDARs and GABARs in the healthy primary motor cortex (M1) measuring plasticity with MEPs. Serial reaction time task (SRTT) data was also collected before and after rTMS as a neurobehavioral outcome measure of motor learning to assess functional integration of plasticity. In line with foundational animal work, we hypothesized that 10-Hz rTMS works through both NMDAR-dependent LTP *and* GABAergic reduction. We predicted, therefore, that relative to placebo, 1) NMDAR agonist DCS would enhance MEPs (as observed previously with a 300-pulse 10-Hz protocol), 2) adding NMDAR antagonist (DCS + dextromethorphan) would block or “knock down” this effect, and that 3) GABAR agonism (lorazepam) would produce relatively increased MEP amplitudes *after* rTMS based on the rationale that *before* rTMS, lorazepam would inhibit MEPs; however if rTMS removed GABARs, lorazepam would then have less receptors to act on, producing less inhibition. We predicted neurobehavioral measures to parallel neurophysiology outcomes, with SRTT response times decreasing after rTMS in the DCS condition and in the lorazepam condition, but not in the “knockdown” dextromethorphan condition.

## Methods

### Ethics approval and consent

Six healthy, right-handed participants (four female) aged 21–51 (29 ± 11.2) with no brain disorders or current use of psychotropic medications provided informed consent and completed a double-blind randomized study with four crossover drug arms (24 experimental subject visits). All study components were approved by the Butler Hospital Institutional Review Board (reference #: 202009-001) and performed in accordance with the relevant guidelines. An online randomizer was used to generate random sequence of drugs for participants. All members of the research team, including investigators, data analysts, and TMS administrators were blinded until data analysis was complete. Exclusion criteria were TMS contraindications of safety concerns, such as implanted metal in the skull and elevated seizure risk. No participants were excluded from initial recruitment. In each visit, participants received a single dose of one of the following identical capsules: placebo (PBO, sucrose), d-cycloserine (DCS, 100 mg), dextromethorphan (DXM, 150 mg) + d-cycloserine (DCS, 100 mg), or lorazepam (LZP, 2.5 mg). We note that this is a high dose of benzodiazepine, but represents the dose used in prior literature with LZP and TMS, as reviewed previously [17]. Rationale for a 4-arm study rather than separate 2-arm studies was to compare the relative contribution of each receptor directly on rTMS within subjects. Direct comparisons of each receptor / modulator would be limited if each experiment were done separately. For example, if GABAR agonism has the predicted effect, but NMDAR agonism is not tested alongside and within subject, we would not know whether individuals have NMDAR or GABAR-mediated mechanisms as our hypothesis predicts.

### Transcranial magnetic stimulation (TMS)

**Fig. 1:**
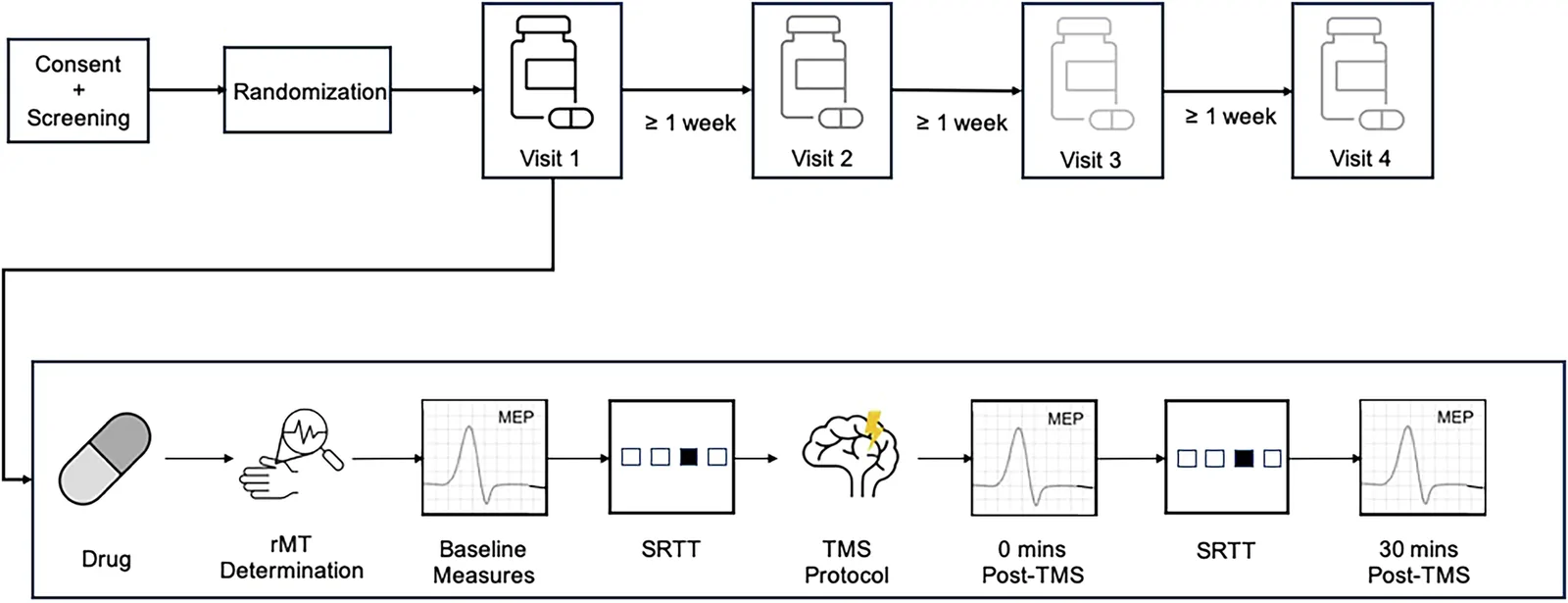
Experimental design. Top: Overview of full experiment with four pill icons representing four drug conditions in randomized order. Bottom: Overview of TMS visit. rMT = resting motor threshold, MEP = motor evoked potential, SRTT = serial reaction time task, Brain icon = 3000 pulses of 10-Hz repetitive transcranial magnetic stimulation with 4 s train duration and 26 s intertrain interval.

All TMS was done at the left M1 using the PowerMAG EEG100 power unit and PMD70-pCool Coil (Mag&More, Germany). At each TMS research visit, we obtained resting motor thresholds (rMT), defined as the lowest stimulator intensity to elicit ≥ 5/10 MEPs of peak-to-peak MEP amplitude of at least 50 μV to determine stimulation intensity for each participant [18]. MEPs were then recorded from the right first dorsal interosseous (FDI) muscle with surface electromyography (EMG) electrodes (Cardinal Health, USA) upon stimulating the left M1 “hotspot.” Single pulse (SP) TMS-induced MEPs were collected before and after 10-Hz rTMS at each study visit. All pulses were kept within 0.5 mm of target. Optimal coil orientation, defined as the orientation producing the highest MEP amplitudes, was determined physiologically and maintained with neuronavigation on a template brain without MRI. Neuronavigation was accomplished with Brainsight 2 (Rogue Research, Quebec, Canada). Study visit procedures are shown in Fig. 1. At each timepoint, we collected 4 bins of 10 MEPs for a total of 40 MEPs, induced by SP at 120% rMT with 4–7 s jitter [5]. Ten MEPs have been previously shown to be sufficient in producing consistent between-session MEP amplitudes in healthy participants [19]. 10-Hz rTMS was administered two hours after drug administration at peak drug bioavailability to the FDI hotspot (3000 pulses, 4 s on/26 s off) at 80% rMT – preferred over higher intensities to reduce seizure risk with M1 stimulation.

### Electromyography (EMG)

MEPs were measured from the right FDI muscle using disposable silver chloride EMG electrodes (Cardinal Health, Inc., United States). We used the 1902 isolated pre-amplifier and Micro 1401 (Cambridge Electronic Design Limited, United Kingdom) to amplify and filter raw EMG data. The EMG signals were sampled, and peak-to-peak amplitudes were calculated using Signal software (Cambridge Electronic Design Limited, United Kingdom). Background noise was continuously monitored.

### Serial reaction time task (SRTT)

To measure the behavioral motor learning changes related to rTMS-induced plasticity, we used a 12-item SRTT administered via MATLab. Participants placed the four fingers of their dominant hand (i.e., index, middle, ring, and pinky fingers) on the “C,” “V,” “B,” and “N” keys of a standard computer keyboard. These keys corresponded to four squares on the computer monitor representing positions 1, 2, 3, and 4. For example, when the right-most square appeared black, individuals had to press the “N” key representing position 4. The task used eight different training sequences and each participant practiced a different training sequence for each visit. Participants repeated each training sequence (T) six times consecutively and performed two 12-item random (R) key presses before and after (i.e., R-T-T-T-T-T-T-R) or before the six repetitions (i.e., R-R-T-T-T-T-T-T). All individuals executed a total of 96 key presses (= 12-item training sequence × 6 repetitions + 12-item random key presses × 2) before and after rTMS during each visit.

### Procedure

All experiments were done in the presence of drug to isolate the effect of receptor modulation on 10-Hz rTMS (Fig. 1). Following drug administration, TMS safety screening questionnaires, and rMT determination (~ 1 h), we measured baseline motor cortical excitability with 40 MEPs, then administered the 3000-pulse protocol. Baseline measures were repeated, with 40 total MEPs measured immediately (0-min), and 30 min (30 min) after 10-Hz rTMS. SRTT was administered immediately before 10-Hz rTMS and 15 min after. Tolerability and effectiveness of blind was evaluated after each visit. Participants reported an average rating of 13.9/100 tolerability (100 being most painful) and correctly guessed which drug they received 42% of the time with 69% confidence in their choices.

### Data analysis

All MEP data were analyzed with R software (version 4.2, R Core Team, Vienna, Austria). Linear Mixed Model (LMM) using the lme4 package was used to determine whether rTMS enhanced MEPs within each drug condition. Raw MEP data from baseline and 30-min post rTMS was analyzed with fixed effect of time and random effect of subject. Analysis was focused on 30-min post rTMS as maximum plasticity was observed at this time point previously (Brown et al., [4]). To compare between drug conditions, we normalized post rTMS MEP amplitudes to total average baseline MEPs within each drug condition for each participant. No outliers or additional transformations were performed. Within 30-min post rTMS, linear mixed model (LMM; lme4) was used to analyze effect of fixed variable drug with random effect of subject. With 6 subjects, 40 MEPs collected per subject, and 4 drug conditions, a total of 960 datapoints were included in the LMM. We did not correct for multiple comparisons as this was a pilot study with a priori hypotheses with previously established separate hypotheses for each placebo v. active drug comparisons. We also used permutation analysis, a test with no inherent statistical assumptions, to determine potential differences between degree of change from baseline to 30-min post rTMS with placebo versus each active drug condition respectively. Analysis was done using sample means with 10,000 permutations for each test. Locally Weighted Scatterplot Smoothing (LOWESS) was used for visualization, calculated in R with a window of 0.2 using the base “stats” package.

For SRTT, we compared differences in mean RT (in seconds) for a correct key press between drug conditions. The RT data were statistically analyzed using SPSS software (Version 28.0.0.0, IBM, United States). Outliers were detected using SPSS based on the interquartile range (IQR). We excluded data points if they fell below Q1 - 1.5 × IQR or above Q3 + 1.5 × IQR. Outliers were removed due to variability observed as result of poor attention and/or sedative drug effects. Normality of the data was verified using Shapiro-Wilk test (*p* > 0.05). Repeated measures ANOVA was used to determine a significant main effect of repeated SRTT trials and included comparison of drug and trial * drug interaction. To compare rTMS effects on overall performance within drug condition, Wilcoxon Signed rank test was used to compare mean RT averaged across pre-rTMS trials to mean RT averaged across post-rTMS trials.

## Results

### MEPs

Corticomotor excitability has not previously been tested with the 3000-pulse protocol. Accordingly, we sought to determine the effect size of potential differences induced by this protocol (i.e., with placebo) in the presence of NMDAR and GABAR modulators to understand the synaptic mechanism of rTMS protocols with clinically-relevant parameters.

**Fig. 2:**
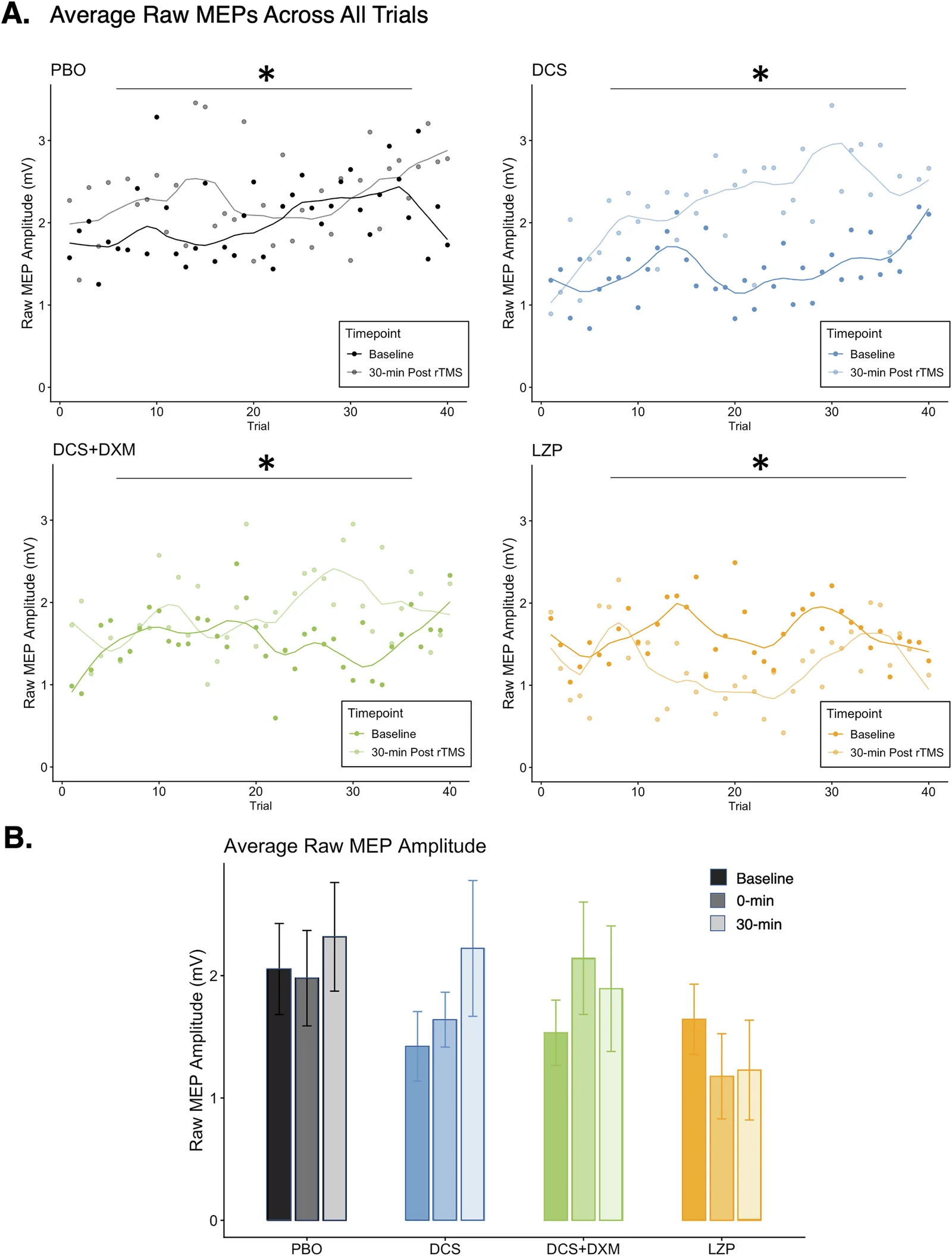
Pre- and post-rTMS MEPs by each drug condition. **A.** Average Raw Motor-Evoked Potentials Over All Trials. Each point represents an average of 6 participants. Dark line is baseline, light line is 30-min Post rTMS. Solid lines represent Locally Weighted Scatterplot Smoothing (LOWESS) with a window of 0.2. **B.** Average Raw MEPs at each timepoint by drug. MEP = motor-evoked potential, BL = baseline, PBO = placebo, DCS = d-cycloserine, DXM = dextromethorphan, LZP = lorazepam.

Figure 2 portrays raw subject data. LMM analysis within each drug condition revealed a significant enhancement of MEPs 30-min post-rTMS relative to baseline for PBO (df = 473, t = 2.31, p = 0.021), DCS (df = 473, t = 6.62, p <0.001) and DCS + DMO (df = 473, t = 3.48, p = 0.001). The LZP condition demonstrated a significant reduction in MEP amplitude at 30-min post-rTMS (df = 473, t = −4.737, p( <0.001). Upon testing to confirm the model met normality and homoscedasticity, Shapiro-wilk test and Levene test revealed violations of assumptions (p < 0.001). Therefore, a permutation analysis was conducted and revealed a significant increase in raw MEP amplitudes from baseline to 30-min post rTMS for DCS relative to PBO (p = 0.008) and a significant decrease with LZP (p = 0.0003). There was no difference between degree of MEP change between PBO v. DCS + DMO (p = 0.613). Mean and standard error for each group was as follows: PBO (2.31 ± 0.108), DCS (2.22 ± 0.125), DCS + DXM (1.89 ± 0.106), LZP (1.23 ± 0.084).

**Fig. 3:**
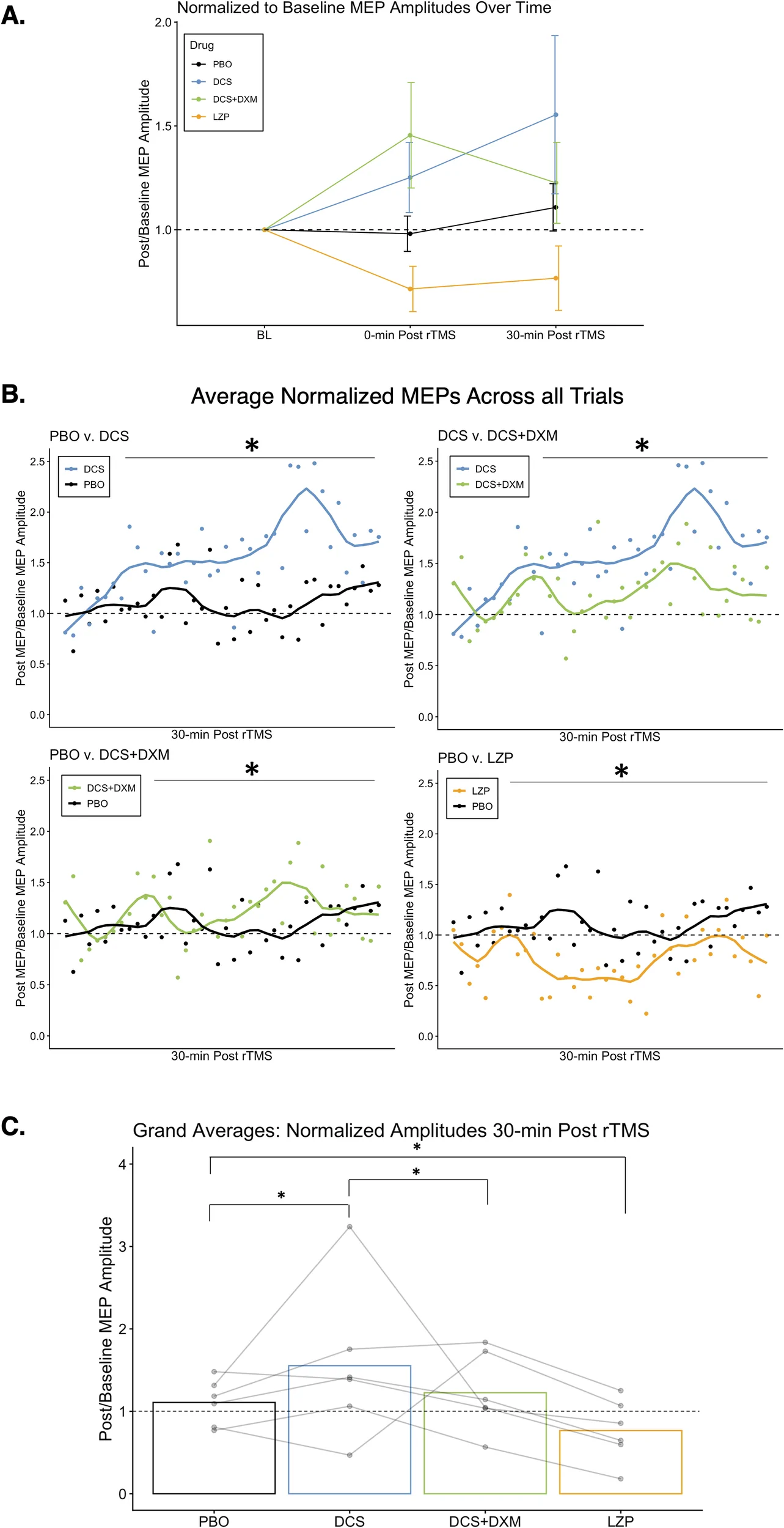
Effect of receptor modulation on rTMS-induced plasticity. Normalized data **A**. Average Normalized to Baseline (dashed line) Motor-Evoked Potentials Over BL, 0-min post rTMS, and 30-min post rTMS. Error bars represent standard error of the mean of 6 participants. **B.** Average normalized to Baseline (dashed line) at 30-min post rTMS over all trials. Each point represents an average of 6 participants. Solid lines represent Locally Weighted Scatterplot Smoothing (LOWESS) with a window of 0.2. **C.** Grand average normalized to baseline at 30-min post rTMS. Gray lines represent individual participant. MEP = motor-evoked potential, BL = baseline, PBO = placebo, DCS = d-cycloserine, DXM = dextromethorphan, LZP = lorazepam.

Descriptive results are presented in Fig. 3A. Individual trial data are illustrated in Fig. 3B with each a priori drug comparison at 30-min post-rTMS. LMM analysis with fixed effect of drug and random effect of subject on MEP amplitude relative to PBO indicated significant effect of DCS (df = 951, t = 5.52, p < 0.001) and LZP (df = 951, t = −4.31, p < 0.001), while no significant effect of DCS + DXM (df=951, t = 1.50, p = 0.135). Compared to a modest increase with PBO after rTMS, we see a greater increase with DCS in most subjects (mean difference = 0.437), being mitigated with DXM. Permutation analysis (Fig. 3C) produced consistent results, demonstrating differences in the degree of MEP change from baseline to 30-min post rTMS between PBO v. DCS (p = 0.0001), PBO v. LZP (p = 0.0003) and DCS v. DCS + DMO (p = 0.0060). PBO v. DCS + DMO was not statistically different (p = 0.184). Mean and standard error for each group was as follows: PBO (1.11 ± 0.043), DCS (1.54 ± 0.085), DCS + DXM (1.23 ± 0.055), LZP (0.767 ± 0.045).

### SRTT

**Fig. 4:**
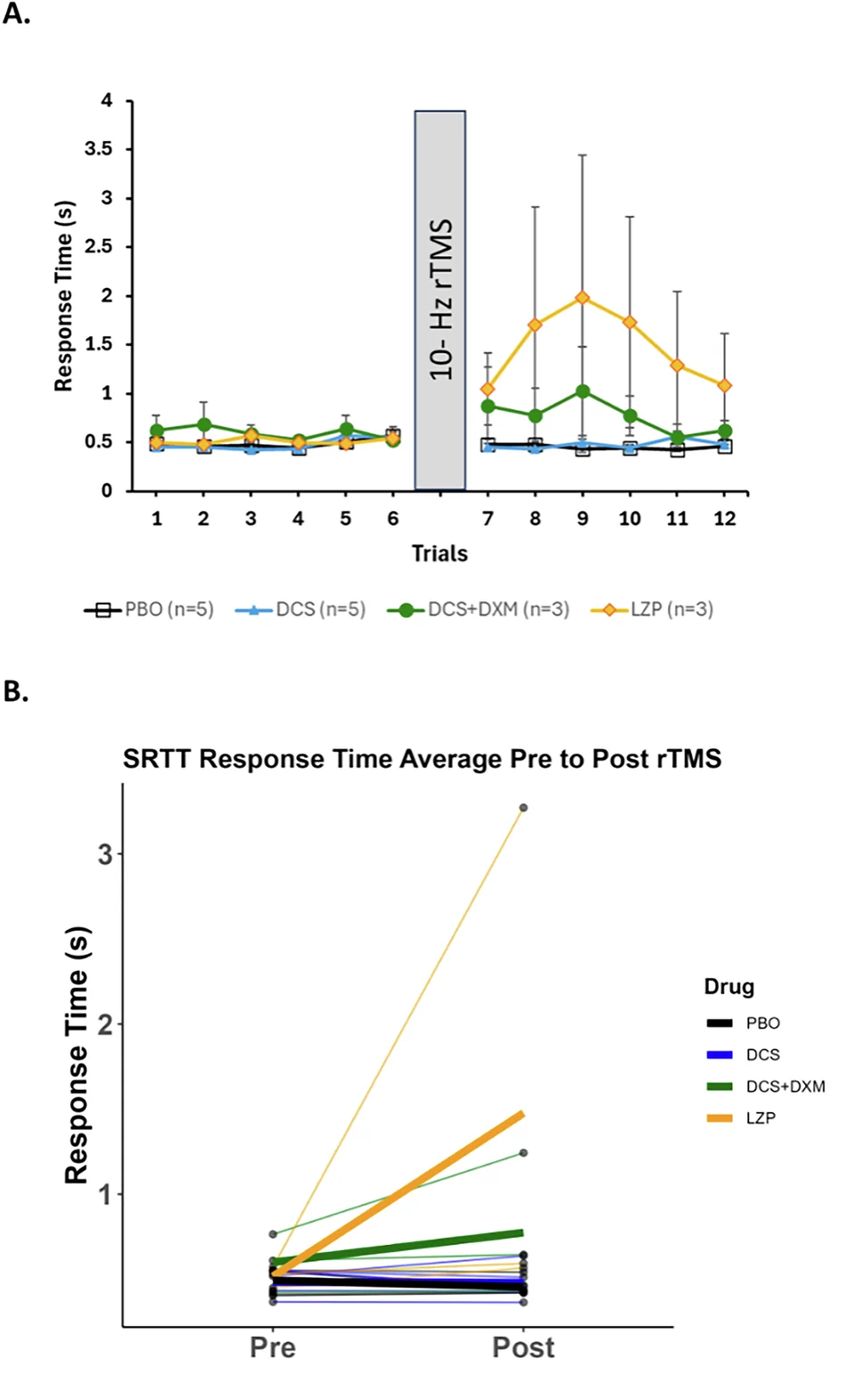
Serial Reaction Time Task. **A** Trials 1 to 6 represent pre-rTMS SRTT performance, and trials 7 to 12 represent post-rTMS performance. Error bars: standard error of the mean. **B** Response time averaged across pre-rTMS trials and post-rTMS trials by subject. Bolded lines represent group averages. rTMS = repetitive transcranial magnetic stimulation; SRTT = serial reaction time task; PBO = placebo, DCS = d-cycloserine, DXM = dextromethorphan, LZP = lorazepam.

We included SRTT in our experiment to evaluate the relationship of physiologic observations with functionally integrated neurobehavioral responses. In this assessment, we hoped to gain insights into a protocol that would neither over nor under train participants to potentially observe differences in either direction between TMS + receptor modulation (i.e., drug conditions). The SRTT has been reported to capture implicit motor learning [20], and has proven modifiable by rTMS [21]. We assessed learning within each condition over trials, including before and after rTMS, and between conditions by comparing reaction time averaged within each trial of 12 sequences. In this preliminary assessment, trend-level effects were not statistically different for trial (Fig. 4A; F(1.573, 18.876) = 3.455, p = 0.062), drug (F(3, 12) = 3.118, p = 0.066) or trial*drug interaction ((F(4.719, 18.876) = 2.462, p = 0.073). We found no drug effect on RTs pre-rTMS (trials 1–6, F(3, 12) = 0.349, p = 0.791) nor post-rTMS (trials 7–12, F(3, 12) = 2.615, p = 0.099). We also found no significant drug effect on offline learning, defined as degree of change from trial 6 to 7 (F(3, 12) = 1.920, p = 0.180). To examine the effect of rTMS with placebo, and of each receptor modulator, we analyzed within subjects averaging pre- and post-rTMS trials together with Wilcoxon Signed Rank test (Fig. 4B): PBO (Pre: 0.493, Post: 0.456; r = -0.31, p = 0.438), DCS (Pre = 0.484, Post = 0.480; r = -0.20, p = 0.625), DCS + DXM (Pre = 0.600, Post = 0.774; r = -0.47, p = 0.250), LZP (Pre = 0.517, Post = 1.48; r = -0.47, p = 0.250).

## Discussion

This study was the first direct examination of the relative contributions of NMDAR v. GABAR in rTMS-induced plasticity. While preliminary, our results appear to support our hypothesis that NMDA receptors significantly contribute to 10-Hz rTMS effects by both enhancing and rescuing motor cortex excitability. Contrary to our original hypothesis that 10-Hz induces both LTP and GABAerigic reduction based on animal findings from Vlachos et al. [2] and Lenz et al. [3], our results in the LZP condition did not support the latter mechanism. Taken together, the data suggest an LTP-like mechanisms for 10-Hz rTMS.

Our aim is to understand how clinical rTMS produces lasting therapeutic effects at a synaptic level, which we consider essential to optimizing rTMS clinical outcomes. This study was also the first to measure plasticity induced by the clinically derived 3000-pulse protocol with a 4 s/26 s duty cycle [15, 16]. Assessing this specific protocol is essential because even small changes in TMS parameters can produce mitigated or even opposing results [12]. For example, the first study to demonstrate pharmacologic augmentation of 10-Hz rTMS utilized a motor physiology protocol of 300 pulses [4] because it produced facilitation over time with 1.5 s train duration and 58.5 s intertrain interval, but caused *inhibition* with a 5/55 s duty cycle [13]. Our results provide key insight that GABAR reduction may not be involved in the mechanism of 10-Hz rTMS, while LTP-like mechanisms are supported with further evidence for the specificity of NMDAR contributions. Regarding feasibility, the four-arm crossover design allowing for direct comparison of receptor modulating drug effects was completed by all participants. However, we learned that the high dose of lorazepam (2.5 mg) and to a lesser degree, dextromethorphan (150 mg), produced drowsiness in three out of six subjects sufficient to impair performance on our neurobehavioral (SRTT) task, indicating a smaller dose may be needed. Given the confounding effect of sedation observed in two conditions and further reduced sample size, we present SRTT data primarily to demonstrate feasibility and guide similar experimental approaches. The knowledge gained from our experiments must be considered in context of several important limitations addressed below.

First, our preliminary results are limited by the small sample size of six subjects (24 experimental visits). Many of the seminal mechanistic TMS studies have had this same challenge (and many have panned out over time and replication) [11, 17, 22, 23]. Though our LMM analyses produced significant results in line with our hypotheses of NMDA-mediated facilitation, the model did not meet normality and homoscedasticity assumptions and likely reflect an inflated type 1 error as result of our sample size. We therefore used a permutation analysis that detected a significant DCS-induced facilitation and LZP-induced reduction in motor plasticity. A well-powered replication experiment is needed to make conclusions about the mechanism of this clinically based TMS protocol.

Second, TMS-induced plasticity in the human motor cortex may differ from plasticity at the dorsolateral prefrontal cortex – the stimulation site for the treatment of depression. The M1 is, by far, the most common site assessed with pharmaco-rTMS plasticity studies due to the convenience of obtaining MEPs [11]. However, regional differences in cortical excitability call into question the translatability of corticomotor findings to other cortical regions [24–29]. M1 experiments also tend to use a lower intensity (80% of rMT), as we did, to minimize risk of inducing seizure. As with other parameter changes, we cannot assume these produce equivalent molecular changes to the clinical 120% or rTMS protocols. Comparisons between protocol intensity are further complicated by the fact that rMT represents a proxy for induced electrical fields, which depend on additional variables such as coil manufacturer, coil orientation, and variations in brain anatomy [30]. Though 10-Hz rTMS continues to be one of the most used clinical protocols to treat depression, it is important to note parameter selection varies widely in clinical settings, particularly with the rise of accelerated protocols. Changes in TMS parameters have been shown to even reverse direction of modulatory effects [14]. Some evidence suggests different, specifically lower intensities are more potent inducers of plasticity [31, 32], but the reason for these results is unknown. Future studies characterizing TMS parameter space are critical to narrow the gap in clinical translation. It is also important to note that MEPs as a measure represent net corticospinal excitability. Modeling suggests that TMS- induced e-fields may exert differential effects on somatic and dendritic segments, as well as distinct neuron groups [3]. MEP results cannot parse out the individual contributions to the summative output ultimately measured, and therefore specific GABA-ergic contributions may be present but not captured.

Third, synaptic plasticity differs between healthy and depressed brains. Decrements in the products of synaptic plasticity (AMPAR gene expression, PSD-95, and synapse numbers) have been observed in post-mortem studies of MDD v. healthy controls [33–35] corresponding with reduced excitability observed in TMS-EEG assessments [36]. Post-mortem reductions in NMDAR subunits, NR2A and NR2B, in MDD patients v. healthy controls could account for decreased plasticity. Interestingly, the NR1 subunit remained unchanged in depressed subjects [34], and it is this subunit that contains the DCS binding site. This could explain why DCS was effective in a clinical trial for depression [37], and why it rescued rTMS-induced excitability in depressed brains near the level of healthy controls [38].

Finally, although pharmacology in humans allows for molecular manipulation of receptors, observations from humans are merely an extrapolation of what occurs during LTP. Such changes include an NMDAR-dependent increase in synaptic transmission, specific increase in synaptic expression of GluA1 receptors (a subtype of AMPA receptor), an increase in spine size from scaffolding proteins and actin cytoskeleton, and involvement proteins like CaMKII and PSD-95 (among many other associated findings) [1]. Therefore, future experiments might include duplicating experiments in animals to look for these markers. DCS was recently shown to enhance LTP in rat hippocampal slices using electrical stimulation [39]. Interestingly, a more robust LTP protocol allowed for only a modest increase, whereas a weaker LTP protocol allowed for greater enhancement from DCS. This may explain why DCS has occluded iTBS [40, 41] but not 10-Hz rTMS [4, 42, 43], as suggested previously [44].

Positive allosteric modulation of GABA _A_ receptors is known to hyperpolarize neurons through the opening of chloride channels and has been demonstrated to decrease MEPs across a multitude of TMS-motor physiology studies [17]. By reducing post-synaptic signaling via GABA_A_ receptor upregulation, LZP may push motor plasticity towards an LTD-like state. If 10-Hz rTMS involves both excitatory and inhibitory neuron involvement, LZP may augment ongoing GABA-ergic processes such as inhibitory interneuron recruitment during and immediately following repetitive stimulation when network activity is increased. Teo et al., [45] demonstrated increased global inhibition as measured by short interval cortical inhibition (SICI) following the same 2.5 dose of LZP combined with practice-dependent plasticity. Though motor practice and rTMS-induced plasticity are not the same, prior studies also suggest plasticity reduction due to nonspecific GABAA activation by LZP. Interestingly, those participants demonstrated improved motor performance despite LZP and enhanced SICI, perhaps attributable to distributed network involvement of plasticity mechanisms contributing to overall learning. Understanding the relevance of GABA agonists in the context of plasticity processes will be key in optimizing TMS in clinical settings, where concomitant benzodiazepine use remains common but its effect unclear.

Lenz and colleagues found that 10-Hz magnetic stimulation reduced GABAR expression, although the pulse number and duty cycle differed [3]. If our clinical protocols produce this effect, the implications on making TMS more effective would change drastically from the current paradigm of enhancing LTP-like effects. Given the risks of a GABAR antagonist (i.e., flumazenil), we sought to test the effects of 10-Hz rTMS on GABARs with an agonist, focusing on the relative post/pre change. We reasoned that while baseline MEP amplitude should be lower with GABAR agonist present due to global shifts in network excitability [46, 47], rTMS-induced removal of GABARs should induce relatively higher MEP amplitudes, since there would be less GABARs for LZP to act on. However, several challenges to this approach should be considered. First, if we had seen an increase, it would be difficult to entangle from LTP-like effects, though we did not face this challenge. Second, lorazepam concentrations peak around 2 h with a 12 h half-life [48], and since individuals will have varying pharmacokinetics, it is possible that some may have a delayed time-to-peak which could result in higher levels of GABAR agonist after rTMS at the time of measurement, thus lowering post-rTMS MEPs.

Arguably one of the most important innovations in the field of TMS is the finding that DCS combined with iTBS produced more than two-fold clinical improvement in a randomized controlled trial [37]. This is the only such clinical trial to date utilizing acute dosing of pharmacologic augmentation, but it represents impressive potential of leverage mechanistic knowledge to augment underlying TMS mechanisms. Neurophysiology studies suggest that other pharmacologic augmentation approaches may also be successful [11]. Well-powered studies examining TMS mechanisms with clinically relevant protocols are necessary to further optimize our protocols for depression and other brain-related disorders. Our pilot study combined a clinically based TMS protocol with multi-receptor pharmacologic augmentation and plasticity-detecting neurophysiology to inform approach for future clinical translation. Future protocols need to move towards our populations, cortical targets, and TMS protocols of interest to avoid inaccurate conclusions and misattributions.

## Supplementary information

Supplemental Tables

## Data Availability

The data that support the findings of this study are available from the corresponding author jbrown@mclean.harvard.edu upon request.

## References

[1] Brown JC, Higgins ES, George MS. Synaptic plasticity 101: the story of the ampa receptor for the brain stimulation practitioner. Neuromodulation. 2022;25:1289–98. 35088731 10.1016/j.neurom.2021.09.003 PMC10479373

[2] Vlachos A, Muller-Dahlhaus F, Rosskopp J, Lenz M, Ziemann U, Deller T. Repetitive magnetic stimulation induces functional and structural plasticity of excitatory postsynapses in mouse organotypic hippocampal slice cultures. J Neurosci. 2012;32:17514–23. 23197741 10.1523/JNEUROSCI.0409-12.2012 PMC6621866

[3] Lenz M, Galanis C, Muller-Dahlhaus F, Opitz A, Wierenga CJ, Szabo G, et al. Repetitive magnetic stimulation induces plasticity of inhibitory synapses. Nat Commun. 2016;7:10020. 26743822 10.1038/ncomms10020 PMC4729863

[4] Brown JC, DeVries WH, Korte JE, Sahlem GL, Bonilha L, Short EB, et al. NMDA receptor partial agonist, d-cycloserine, enhances 10 Hz rTMS-induced motor plasticity, suggesting long-term potentiation (LTP) as underlying mechanism. Brain Stimul. 2020;13:530–2. 32289670 10.1016/j.brs.2020.01.005 PMC7224691

[5] Kweon J, Vigne M, Jones R, George MS, Carpenter LL, Brown JC. A replication study of NMDA receptor agonism sufficiency to enhance 10-Hz rTMS-induced motor cortex plasticity. Brain Stimul. 2022;15:1372–4. 36180040 10.1016/j.brs.2022.09.014 PMC12599535

[6] Brown JC, Yuan S, DeVries WH, Armstrong NM, Korte JE, Sahlem GL, et al. NMDA-receptor agonist reveals LTP-like properties of 10-Hz rTMS in the human motor cortex. Brain Stimul. 2021;14:619–21. 33794339 10.1016/j.brs.2021.03.016 PMC8164996

[7] Kweon J, Vigne MM, Jones RN, Carpenter LL, Brown JC. Practice makes plasticity: 10-Hz rTMS enhances LTP-like plasticity in musicians and athletes. Front Neural Circuits. 2023;17:1124221. 37025991 10.3389/fncir.2023.1124221 PMC10070804

[8] Vigne M, Kweon J, Sharma P, Greenberg BD, Carpenter LL, Brown JC. Chronic caffeine consumption curbs rTMS-induced plasticity. Front Psychiatry. 2023;14:1137681. 36911138 10.3389/fpsyt.2023.1137681 PMC9993245

[9] Heidegger T, Krakow K, Ziemann U. Effects of antiepileptic drugs on associative LTP-like plasticity in human motor cortex. Eur J Neurosci. 2010;32:1215–22. 20726885 10.1111/j.1460-9568.2010.07375.x

[10] McDonnell MN, Orekhov Y, Ziemann U. Suppression of LTP-like plasticity in human motor cortex by the GABAB receptor agonist baclofen. Exp Brain Res. 2007;180:181–6. 17351767 10.1007/s00221-006-0849-0

[11] Sohn MN, Brown JC, Sharma P, Ziemann U, McGirr A. Pharmacological adjuncts and transcranial magnetic stimulation-induced synaptic plasticity: a systematic review. J Psychiatry Neurosci. 2024;49:E59–E76. 38359933 10.1503/jpn.230090 PMC10890793

[12] Caulfield KA, Brown JC. The problem and potential of tms’ infinite parameter space: a targeted review and road map forward. Front Psychiatry. 2022;13:867091. 35619619 10.3389/fpsyt.2022.867091 PMC9127062

[13] Jung SH, Shin JE, Jeong YS, Shin HI. Changes in motor cortical excitability induced by high-frequency repetitive transcranial magnetic stimulation of different stimulation durations. Clin Neurophysiol. 2008;119:71–9. 18039593 10.1016/j.clinph.2007.09.124

[14] Gamboa OL, Antal A, Moliadze V, Paulus W. Simply longer is not better: reversal of theta burst after-effect with prolonged stimulation. Exp Brain Res. 2010;204:181–7. 20567808 10.1007/s00221-010-2293-4 PMC2892066

[15] O’Reardon JP, Solvason HB, Janicak PG, Sampson S, Isenberg KE, Nahas Z, et al. Efficacy and safety of transcranial magnetic stimulation in the acute treatment of major depression: a multisite randomized controlled trial. Biol Psychiatry. 2007;62:1208–16. 17573044 10.1016/j.biopsych.2007.01.018

[16] George MS, Lisanby SH, Avery D, McDonald WM, Durkalski V, Pavlicova M, et al. Daily left prefrontal transcranial magnetic stimulation therapy for major depressive disorder: a sham-controlled randomized trial. Arch Gen Psychiatry. 2010;67:507–16. 20439832 10.1001/archgenpsychiatry.2010.46

[17] Ziemann U, Reis J, Schwenkreis P, Rosanova M, Strafella A, Badawy R, et al. TMS and drugs revisited 2014. Clin Neurophysiol. 2015;126:1847–68. 25534482 10.1016/j.clinph.2014.08.028

[18] Rossini PM, Burke D, Chen R, Cohen LG, Daskalakis Z, Di Iorio R, et al. Non-invasive electrical and magnetic stimulation of the brain, spinal cord, roots and peripheral nerves: basic principles and procedures for routine clinical and research application. an updated report from an I.F.C.N. committee. Clin Neurophysiol. 2015;126:1071–107. 25797650 10.1016/j.clinph.2015.02.001 PMC6350257

[19] Cavaleri R, Schabrun SM, Chipchase LS. The number of stimuli required to reliably assess corticomotor excitability and primary motor cortical representations using transcranial magnetic stimulation (TMS): a systematic review and meta-analysis. Syst Rev. 2017;6:48. 28264713 10.1186/s13643-017-0440-8 PMC5340029

[20] Robertson EM. The serial reaction time task: implicit motor skill learning?. J Neurosci. 2007;27:10073–5. 17881512 10.1523/JNEUROSCI.2747-07.2007 PMC6672677

[21] Szucs-Bencze L, Vekony T, Pesthy O, Szabo N, Kincses TZ, Turi Z, et al. Modulating visuomotor sequence learning by repetitive transcranial magnetic stimulation: what do we know so far?. J Intell. 2023;11:201. 37888433 10.3390/jintelligence11100201 PMC10607545

[22] Huang YZ, Chen RS, Rothwell JC, Wen HY. The after-effect of human theta burst stimulation is NMDA receptor dependent. Clin Neurophysiol. 2007;118:1028–32. 17368094 10.1016/j.clinph.2007.01.021

[23] Huang YZ, Edwards MJ, Rounis E, Bhatia KP, Rothwell JC. Theta burst stimulation of the human motor cortex. Neuron. 2005;45:201–6. 15664172 10.1016/j.neuron.2004.12.033

[24] Casula EP, Tarantino V, Basso D, Arcara G, Marino G, Toffolo GM, et al. Low-frequency rTMS inhibitory effects in the primary motor cortex: Insights from TMS-evoked potentials. Neuroimage. 2014;98:225–32. 24793831 10.1016/j.neuroimage.2014.04.065

[25] Veniero D, Ponzo V, Koch G. Paired associative stimulation enforces the communication between interconnected areas. J Neurosci. 2013;33:13773–83. 23966698 10.1523/JNEUROSCI.1777-13.2013 PMC6618662

[26] Freedberg M, Reeves JA, Hussain SJ, Zaghloul KA, Wassermann EM. Identifying site- and stimulation-specific TMS-evoked EEG potentials using a quantitative cosine similarity metric. PLoS One. 2020;15:e0216185. 31929531 10.1371/journal.pone.0216185 PMC6957143

[27] Kahkonen S, Komssi S, Wilenius J, Ilmoniemi RJ. Prefrontal TMS produces smaller EEG responses than motor-cortex TMS: implications for rTMS treatment in depression. Psychopharmacology. 2005;181:16–20. 15719214 10.1007/s00213-005-2197-3

[28] Lioumis P, Kicic D, Savolainen P, Makela JP, Kahkonen S. Reproducibility of TMS-Evoked EEG responses. Hum Brain Mapp. 2009;30:1387–96. 18537115 10.1002/hbm.20608 PMC6870729

[29] Ozdemir RA, Tadayon E, Boucher P, Sun H, Momi D, Ganglberger W, et al. Cortical responses to noninvasive perturbations enable individual brain fingerprinting. Brain Stimul. 2021;14:391–403. 33588105 10.1016/j.brs.2021.02.005 PMC8108003

[30] Drakaki M, Mathiesen C, Siebner HR, Madsen K, Thielscher A. Database of 25 validated coil models for electric field simulations for TMS. Brain Stimul. 2022;15:697–706. 35490970 10.1016/j.brs.2022.04.017

[31] Chung SW, Rogasch NC, Hoy KE, Sullivan CM, Cash RFH, Fitzgerald PB. Impact of different intensities of intermittent theta burst stimulation on the cortical properties during TMS-EEG and working memory performance. Hum Brain Mapp. 2018;39:783–802. 29124791 10.1002/hbm.23882 PMC6866298

[32] Nettekoven C, Volz LJ, Kutscha M, Pool EM, Rehme AK, Eickhoff SB, et al. Dose-dependent effects of theta burst rTMS on cortical excitability and resting-state connectivity of the human motor system. J Neurosci. 2014;34:6849–59. 24828639 10.1523/JNEUROSCI.4993-13.2014 PMC4019799

[33] Duric V, Banasr M, Stockmeier CA, Simen AA, Newton SS, Overholser JC, et al. Altered expression of synapse and glutamate related genes in post-mortem hippocampus of depressed subjects. Int J Neuropsychopharmacol. 2013;16:69–82. 22339950 10.1017/S1461145712000016 PMC3414647

[34] Feyissa AM, Chandran A, Stockmeier CA, Karolewicz B. Reduced levels of NR2A and NR2B subunits of NMDA receptor and PSD-95 in the prefrontal cortex in major depression. Prog Neuropsychopharmacol Biol Psychiatry. 2009;33:70–5. 18992785 10.1016/j.pnpbp.2008.10.005 PMC2655629

[35] Kang HJ, Voleti B, Hajszan T, Rajkowska G, Stockmeier CA, Licznerski P, et al. Decreased expression of synapse-related genes and loss of synapses in major depressive disorder. Nat Med. 2012;18:1413–7. 22885997 10.1038/nm.2886 PMC3491115

[36] Noda Y, Zomorrodi R, Vila-Rodriguez F, Downar J, Farzan F, Cash RFH, et al. Impaired neuroplasticity in the prefrontal cortex in depression indexed through paired associative stimulation. Depress Anxiety. 2018;35:448–56. 29637656 10.1002/da.22738

[37] Cole J, Sohn MN, Harris AD, Bray SL, Patten SB, McGirr A. Efficacy of adjunctive D-cycloserine to intermittent theta-burst stimulation for major depressive disorder: a randomized clinical trial. JAMA Psychiatry. 2022;79:1153–61. 36223114 10.1001/jamapsychiatry.2022.3255 PMC9557938

[38] Cole J, Selby B, Ismail Z, McGirr A. D-cycloserine normalizes long-term motor plasticity after transcranial magnetic intermittent theta-burst stimulation in major depressive disorder. Clin Neurophysiol. 2021;132:1770–6. 34130243 10.1016/j.clinph.2021.04.002

[39] Vestring S, Dorner A, Scholliers J, Ehrenberger K, Kiss A, Arenz L, et al. D-Cycloserine enhances the bidirectional range of NMDAR-dependent hippocampal synaptic plasticity. Transl Psychiatry. 2024;14:18. 38195548 10.1038/s41398-023-02725-7 PMC10776623

[40] Teo JT, Swayne OB, Rothwell JC. Further evidence for NMDA-dependence of the after-effects of human theta burst stimulation. Clin Neurophysiol. 2007;118:1649–51. 17502166 10.1016/j.clinph.2007.04.010

[41] Selby B, MacMaster FP, Kirton A, McGirr A. d-cycloserine blunts motor cortex facilitation after intermittent theta burst transcranial magnetic stimulation: A double-blind randomized placebo-controlled crossover study. Brain Stimul. 2019;12:1063–5. 30914260 10.1016/j.brs.2019.03.026

[42] Brown JC, Yuan S, DeVries WH, Armstrong NM, Korte JE, Sahlem GL, et al. NMDA-receptor agonist reveals LTP-like properties of 10-Hz rTMS in the human motor cortex. BraStimulation: Basic, Translational, Clin Res Neuromodulation. 2021;14:619–21. 10.1016/j.brs.2021.03.016 PMC8164996 33794339

[43] Kweon J, Vigne M, Jones R, George MS, Carpenter LL, Brown JC. A replication study of NMDA receptor agonism sufficiency to enhance 10-Hz rTMS-induced motor cortex plasticity. BraStimulation: Basic, Translational, Clin Res Neuromodulation. 2022;15:1372–4. 10.1016/j.brs.2022.09.014 PMC12599535 36180040

[44] Brown JC, Higgins ES, George MS. Synaptic plasticity 101: the story of the AMPA receptor for the brain stimulation practitioner. Neuromodulation. 2021;25:1289–98. 35088731 10.1016/j.neurom.2021.09.003 PMC10479373

[45] Teo JT, Terranova C, Swayne O, Greenwood RJ, Rothwell JC. Differing effects of intracortical circuits on plasticity. Exp Brain Res. 2009;193:555–63. 19048237 10.1007/s00221-008-1658-4 PMC3019102

[46] Di Lazzaro V, Oliviero A, Meglio M, Cioni B, Tamburrini G, Tonali P, et al. Direct demonstration of the effect of lorazepam on the excitability of the human motor cortex. Clin Neurophysiol. 2000;111:794–9. 10802448 10.1016/s1388-2457(99)00314-4

[47] Di Lazzaro V, Oliviero A, Saturno E, Dileone M, Pilato F, Nardone R, et al. Effects of lorazepam on short latency afferent inhibition and short latency intracortical inhibition in humans. J Physiol. 2005;564:661–8. 15718269 10.1113/jphysiol.2004.061747 PMC1464438

[48] Ghiasi, N, RK Bhansali, and R Marwaha, *Lorazepam*, in *StatPearls*. 2024: Treasure Island (FL). 30422485

