# Supplementary material for: Exploration of NMDA and GABA receptor-mediated plasticity induced by 10-Hz repetitive transcranial magnetic stimulation
Source: Transl Psychiatry. 2026 Jun 17;16:430. doi: 10.1038/s41398-026-04159-3 (PMC13507194; doi:10.1038/s41398-026-04159-3)
Supplement: Supplementary file 1 [file 41398_2026_4159_MOESM1_ESM.docx]

| Fixed Effects | | | | | | |
| --- | --- | --- | --- | --- | --- | --- |
|  | | Est/Beta | | SE | t | P |
| **Condition: PBO** | | | | | | |
| Intercept | | 2.05 | | .400 | 5.14 | .003 |
| 0-min v. 30-min | | .131 | | .057 | 2.31 | .021 |
| **Condition: DCS** | | | | | | |
| Intercept | | 1.43 | | .408 | 3.517 | .016 |
| 0-min v. 30-min | | .394 | | .059 | 6.66 | <.0001 |
| **Condition: DCS + DMO** | | | | | | |
| Intercept | | 1.53 | | .376 | 4.08 | .009 |
| 0-min v. 30-min | | .180 | | .052 | 3.48 | .001 |
| **Condition: LZP** | | | | | | |
| Intercept | | 1.64 | | .322 | 5.11 | .003 |
| 0-min v. 30-min | | -.208 | | .044 | -4.74 | <.001 |
| Random Effects | | | | | | |
|  |  | |  | | Variance | S.D. |
| **Condition: PBO** | | | | | | |
| Subject (Intercept) | | | | | .920 | .959 |
| Residual | | | | | 1.55 | 1.24 |
| **Condition: DCS** | | | | | | |
| Subject (Intercept) | | | | | .957 | .978 |
| Residual | | | | | 1.68 | 1.29 |
| **Condition: DCS +DMO** | | | | | | |
| Subject (Intercept) | | | | | .816 | .903 |
| Residual | | | | | 1.29 | 1.13 |
| **Condition: LZP** | | | | | | |
| Subject (Intercept) | | | | | .598 | .773 |
| Residual | | | | | .923 | .961 |

**Supplemental Table 1.** LMM results of raw MEP comparisons between 0-min to 30-min post-rTMS within each drug condition. Analysis with fixed effect of time and random effect of subject.

| Fixed Effects | | | | |
| --- | --- | --- | --- | --- |
|  | Est/Beta | SE | t | p |
| Intercept | 1.11 | .144 | 7.67 | .0001 |
| PBO v. DCS | .437 | .079 | 5.52 | <.0001 |
| PBO v. DCS+DMO | .118 | .079 | 1.50 | .135 |
| PBO v. LZP | -.341 | .079 | -4.31 | <.0001 |
| Random Effects | | | | |
|  | | | Variance | S.D. |
| Subject (Intercept) | | | .106 | .326 |
| Residual | | | .752 | .867 |

**Supplemental Table 2.** LMM results of each *a priori* drug comparison at 30-min post-rTMS. Analysis with fixed effect of drug and random effect of subject on MEP amplitude relative to PBO.

| Comparison | P | Difference in Means |
| --- | --- | --- |
| **Raw MEPs** | | |
| Δ PBO v. Δ DCS | .008 | .524 |
| Δ PBO v. Δ DCS + DMO | .613 | .098 |
| Δ DCS v. Δ DCS + DMO | .026 | -.426 |
| Δ PBO v. Δ LZP | .0003 | -.678 |
| **Normalized MEPs** | | |
| Δ PBO v. Δ DCS | .0001 | .437 |
| Δ PBO v. Δ DCS + DMO | .184 | .118 |
| Δ DCS v. Δ DCS + DMO | .006 | -.319 |
| Δ PBO v. Δ LZP | 0 | -.341 |

**Supplemental Table 3.** Permutation analysis comparing degree of MEP amplitude change from 0-min to 30-min post rTMS between PBO and active drug conditions and DCS v. DCS+DMO to evaluate NMDAR specificity.
